# The number and microlocalization of tumor-associated immune cells are associated with patient's survival time in non-small cell lung cancer

**DOI:** 10.1186/1471-2407-10-220

**Published:** 2010-05-20

**Authors:** Fuqiang Dai, Lunxu Liu, Guowei Che, Nanbin Yu, Qiang Pu, Shangfu Zhang, Junliang Ma, Lin Ma, Zongbing You

**Affiliations:** 1 Department of Thoracic and Cardiovascular Surgery, West China Hospital, Sichuan University, Chengdu 610041, China; 2 Daping Hospital, the Third Military Medical University, Chongqing City, China; 3 The Third People's Hospital of Zigong City, Sichuan Province, China; 4 Departments of Structural & Cellular Biology and Orthopaedic Surgery, Tulane Cancer Center, LCRC, Tulane Center for Aging, Tulane Center for Gene Therapy, Tulane University School of Medicine, New Orleans, LA 70112, USA

## Abstract

**Background:**

Tumor microenvironment is composed of tumor cells, fibroblasts, endothelial cells, and infiltrating immune cells. Tumor-associated immune cells may inhibit or promote tumor growth and progression. This study was conducted to determine whether the number and microlocalization of macrophages, mature dendritic cells and cytotoxic T cells in non-small cell lung cancer are associated with patient's survival time.

**Methods:**

Ninety-nine patients with non-small cell lung cancer (NSCLC) were included in this retrospective study. Paraffin-embedded NSCLC specimens and their clinicopathological data including up to 8-year follow-up information were used. Immunohistochemical staining for CD68 (marker for macrophages), CD83 (marker for mature dendritic cells), and CD8 (marker for cytotoxic T cells) was performed and evaluated in a blinded fashion. The numbers of immune cells in tumor islets and stroma, tumor islets, or tumor stroma were counted under a microscope. Correlation of the cell numbers and patient's survival time was analyzed using the Statistical Package for the Social Sciences (version 13.0).

**Results:**

The numbers of macrophages, mature dendritic cells and cytotoxic T cells were significantly more in the tumor stroma than in the tumor islets. The number of macrophages in the tumor islets was positively associated with patient's survival time, whereas the number of macrophages in the tumor stroma was negatively associated with patient's survival time in both univariate and multivariate analyses. The number of mature dendritic cells in the tumor islets and stroma, tumor islets only, or tumor stroma only was positively associated with patient's survival time in a univariate analysis but not in a multivariate analysis. The number of cytotoxic T cells in the tumor islets and stroma was positively associated with patient's survival time in a univariate analysis but not in a multivariate analysis. The number of cytotoxic T cells in the tumor islets only or stroma only was not associated with patient's survival time.

**Conclusions:**

The number of macrophages in the tumor islets or stroma is an independent predictor of survival time in NSCLC patients. Counting macrophages in the tumor islets or stroma is more useful in predicting patient's survival time than counting mature dendritic cells or cytotoxic T cells.

## Background

Tumor microenvironment is composed of tumor cells, resident cells such as fibroblasts and endothelial cells, and infiltrating cells such as macrophages, dendritic cells, and lymphocytes, as well as products of all these cells such as extracellular matrix, growth factors, cytokines, chemokines, enzymes, and various metabolites [1]. The cross-talk between tumor cells and other tumor-associated cells may lead to either blocking tumor formation or enhancing tumor formation and/or progression. The double-edged-sword nature of many tumor-associated immune cells such as macrophages, dendritic cells, and cytotoxic T cells has been recognized [2-4]. On the one hand, these immune cells may recognize tumor-associated antigen and activate cytotoxic T cells, in order to initiate anti-tumor immune responses. On the other hand, the same immune cells may establish immune tolerance and even promote tumor growth and metastasis through enhancing angiogenesis and invasion of extracellular matrix.

Non-small cell lung cancer (NSCLC) is the most common cause of cancer-related death worldwide. The five-year survival rate is approximately 67% for the patients with stage IA NSCLC after putatively curative surgery [5]. In order to identify new prognostic factors that can guide clinical practice, we have previously found that the number of tumor-associated macrophages (TAMs) in the tumor islets is positively associated with survival time in the patients with NSCLC [6]. Because TAMs are not the only tumor-associated immune cells, in this study we further investigated the prognostic value of mature dendritic cells and cytotoxic T cells in the patients with NSCLC.

## Methods

### Study population

This study was approved by the Institutional Review Board of West China Hospital, Sichuan University. The procedures to obtain human lung cancer tissues and follow-up information are in accordance with the Ethical Principles for Medical Research Involving Human Subjects as formulated in the World Medical Association Declaration of Helsinki (revised in 2008). All specimens were obtained from the archives of formalin-fixed, paraffin-embedded tissue blocks in the Department of Thoracic and Cardiovascular Surgery, West China Hospital, Sichuan University. The lung cancer tissues were collected from surgeries performed from August, 1999 to August, 2001. The patients were followed up until December, 2007, through outpatient visits and/or correspondences to family members. Ninety-nine patients were included in this retrospective study. Histological evaluation was based on the World Health Organization criteria [7]. Tumor stage was evaluated according to the International Union against Cancer TNM classification system [7]. The clinicopathological characteristics were summarized in Table 1.

**Table 1:** Clinicopathological characteristics of patients with non-small cell lung cancer (n = 99)

Variable	N = 99	5-yr survival (%)
Age years median (range)	60 ( 37 - 80)	Overall
Gender (male: female)	80:19	32
Tumor stage:		
I	35	49
II	20	35
III	34	19
IV	10	10
Histology		
Adenocarcinoma	45	22
Squamous cell carcinoma	51	41
Large cell carcinoma	3	33
Tumor grade		
Well differentiation	3	67
Moderate differentiation	48	40
Poor differentiation	29	24
Not recorded	19	20
Lymph node metastasis		
No	59	44
Yes	40	15

Histological criteria for the tumor grading:

Adenocarcinomas:

Well differentiation: glandular lumens are large and similar in sizes, with less cytologic atypia.

Moderate differentiation: glandular lumens vary in sizes, with obvious cytologic atypia.

Poor differentiation: glandular lumens are small or absent, with obvious cytologic atypia and mitosis.

Squamous cell carcinomas:

Well differentiation: ≥ 20% of the tumor islets has pearl formation.

Moderate differentiation: < 20% of the tumor islets has pearl formation.

Poor differentiation: no pearl formation in the tumor islets; only shows intracellular keratinization.

Large cell carcinomas: by definition, are poorly differentiated tumors that lack the cytologic and architectural features of small cell carcinoma, squamous cell carcinoma, or adenocarcinoma.

### Immunohistochemistry

Four-μm thick tissue sections were de-waxed in xylene and rehydrated through graded alcohols. Antigen retrieval was carried out using microwave at middle-to-high temperature for 8 min, low-to-high temperature for 5 min, and then cooled down at room temperature for 20 min. Mouse anti-human CD68 monoclonal antibodies (clone KP1, recognizing macrophages), rabbit anti-human CD8 monoclonal antibodies (clone SP16, recognizing cytotoxic T cells), and streptavidin-peroxidase conjugated secondary antibodies (SP-9002) were obtained from Zhongshan Goldenbridge Biotechnology Co., LTD., Beijing, China. Mouse anti-human CD83 monoclonal antibodies (clone HB15a, recognizing mature dendritic cells) were obtained from Santa Cruz Biotechnology, Inc., Santa Cruz, CA, USA. Diaminobenzidine (DAB) substrate kit was obtained from Dako North America, Inc., Carpinteria, CA, USA. Immunohistochemical staining of individual markers was performed according to the kit manufacturer's instructions. Sections were then counterstained with hematoxylin and mounted in an aqueous mounting medium. Tissue sections previously stained positively were used as positive control, while tissue sections with primary antibodies replaced by phosphate-buffered saline served as negative control. Positive cells showed brown particles on the cellular membrane and/or in the cytoplasm. Under a microscope, five representative high-power fields (× 400 magnification) of the tumor islets and stroma per tissue section were selected and counted for positive cell numbers. The average number of these five high-power fields represented the cell number per high-power field. Evaluation of the stained tissue sections was performed by two investigators who were blinded in regard to the clinicopathological characteristics. An average of the results obtained by the two examiners was used to represent each case.

### Statistical analysis

Statistical analysis was carried out using the Statistical Package for the Social Sciences (SPSS, version 13.0, SPSS Inc., Chicago, IL, USA). For categorical analysis, the median number of cells per high-power field was used as a cut-off point to dichotomize the continuous variables. The Mann-Whitney nonparametric test was used to compare between two groups. In order to assess any potential relationship between the number of immune cells and patient's survival time, the Spearman's rank correlation coefficient (Spearman's rho, or r_s_) was calculated in a univariate analysis. The correlation between the number of immune cells and the clinicopathological characteristics was analyzed using the χ^2 ^test. The Kaplan-Meier survival curves were used to look for correlation between the cell number and patient's survival time, or between the ratio of cells in the tumor islets versus stroma and patient's survival time. Statistical significance was analyzed using the log-rank test. A multivariate Cox proportional hazards model was used to estimate adjusted hazard ratios and 95% confidence intervals (CI) and to identify which variable was an independent prognostic factor. The validity of the proportional hazards assumption was assessed from log (-log [Survival]) curves. For the above comparisons, P < 0.05 was considered statistically significant.

## Results

### Patient characteristics

All of the 99 patients (Table 1) had complete follow-up information and the pathological diagnosis was verified by a pathologist prior to inclusion in this study. The overall cumulative 5-year survival rate was 32%. Patients with stage I carcinomas, squamous cell carcinomas, well-differentiated carcinomas, or carcinomas without lymph node metastasis had better survival rates than the other patients (Table 1).

### Immunohistochemical detection of the tumor-associated immune cells

**Figure 1:**
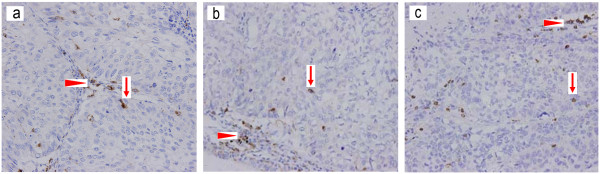
**Immunohistochemical detection of tumor-associated immune cells in non-small cell lung cancer tissues**. (a) Macrophages (marker CD68) stained brown in the tumor islets (arrow) and tumor stroma (arrowhead). (b) Mature dendritic cells (marker CD83) stained brown in the tumor islets (arrow) and tumor stroma (arrowhead). (c) Cytotoxic T cells (marker CD8) stained brown in the tumor islets (arrow) and tumor stroma (arrowhead). Original magnification, × 400.

Macrophages, mature dendritic cells, and cytotoxic T cells were detected by anti-CD68 antibodies, anti-CD83 antibodies, and anti-CD8 antibodies, respectively (Figure 1). These tumor-associated immune cells were located in both the tumor islets and stroma. Under each high-power field (× 400 magnification), the median number of macrophages in the tumor islets and stroma is approximately 15 (Table 2). The median number of macrophages in the tumor islets only is approximately 4. The median number of macrophages in the tumor stroma only is approximately 7, which was significantly more than that in the tumor islets (P = 0.040). The median numbers of mature dendritic cells in the tumor islets and stroma, tumor islets only, or tumor stroma only were approximately 13, 4, or 7, respectively (Table 2). The number of mature dendritic cells in the tumor stroma was significantly more than that in the tumor islets (P = 0.000). The median numbers of CD8+ cytotoxic T cells in the tumor islets and stroma, tumor islets only, or tumor stroma only were approximately 11, 4, or 7, respectively (Table 2). The number of CD8+ cytotoxic T cells in the tumor stroma was significantly more than that in the tumor islets (P = 0.032).

**Table 2:** Correlation between the number and microlocalization of immune cells and patient's survival time by a univariate analysis

Cell type	Islets + Stroma		Islets		Stroma		P (islets versus stroma)
		
	Number	Spearman's rho & P	Number	Spearman's rho & P	Number	Spearman's rho & P	
Macrophages	15.2 (1.8 - 48.4)	r_s _= -0.105 P = 0.300	4.2 (0.2 - 40.6)	r_s _= 0.483 P = 0.000	7.2 (0.4 - 39.6)	r_s _= -0.541 P = 0.000	0.040
Mature dendritic cells	13.2 (1.4 - 137.6)	r_s _= 0.221 P = 0.028	4.2 (0 - 21.4)	r_s _= 0.404 P = 0.001	7.0 (0.2 - 25.4)	r_s _= 0.284 P = 0.045	0.000
CD8+ T cells	10.8 (0.4 - 45.0)	r_s _= 0.297 P = 0.003	4.2 (0 - 66.2)	r_s _= 0.247 P = 0.014	7.2 (0.2 - 71.4)	r_s _= 0.212 P = 0.035	0.032

The number of immune cells represents median (range) per high-power field. The number of immune cells in the tumor islets and stroma (Islets + Stroma) was obtained by combining the number in the tumor islets and the number in the tumor stroma in each case. Then, the numbers of immune cells in the tumor islets and stroma were ranked and the median number was determined. Therefore, the median number of immune cells in the tumor islets and stroma is not a simple sum of the median number in the tumor islets and the median number in the tumor stroma as shown in this table.

### Correlation between the number and microlocalization of immune cells and survival time

The Spearman's rank correlation coefficient (Spearman's rho, or r_s_) was calculated to assess any potential relationship between the tumor-associated immune cells and patient's survival time. We found that the macrophage number in the tumor islets and stroma had no association with patient's survival time (r_s _= -0.105, P = 0.300). The macrophage number in the tumor islets was positively associated with patient's survival (r_s _= 0.483, P = 0.000). In contrast, the macrophage number in the tumor stroma was negatively associated with patient's survival (r_s _= -0.541, P = 0.000) (Table 2). The numbers of mature dendritic cells in the tumor islets and stroma, tumor islets, and tumor stroma were all positively associated with patient's survival time, with r_s _= 0.221 (P = 0.028), 0.404 (P = 0.001), and 0.284 (P = 0.045), respectively. Similarly, the numbers of CD8+ cytotoxic T cells in the tumor islets and stroma, tumor islets, and tumor stroma were also positively associated with patient's survival time, with r_s _= 0.297 (P = 0.003), 0.247 (P = 0.014), and 0.212 (P = 0.035), respectively (Table 2).

**Figure 2:**
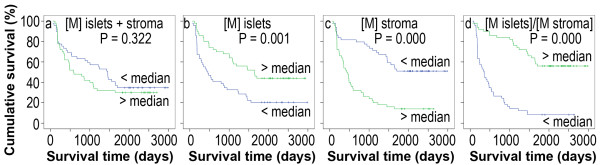
**Kaplan-Meier survival curves demonstrate macrophage number or ratio in correlation to survival**. [M] represents the macrophage number in the tumor islets and stroma (a), tumor islets (b), and tumor stroma (c). (d) The ratio of the macrophage number in the tumor islets "[M islets]" versus the macrophage number in the tumor stroma "[M stroma]".

In order to assess whether there is any value in predicting prognosis, the median number of the tumor-associated immune cells was used as a cut-off point to dichotomize the 99 patients into a group with a cell number above the median and a group with a cell number below the median. We found that the patients with above-the-median macrophage number in the tumor islets and stroma had a similar Kaplan-Meier survival curve, compared to those patients with below-the-median macrophage number in the tumor islets and stroma (P = 0.322, Figure 2a). The patients with above-the-median macrophage number in the tumor islets had a significantly better cumulative survival, compared to the patients with below-the-median macrophage number in the tumor islets (P = 0.001, Figure 2b). In contrast, the patients with above-the-median macrophage number in the tumor stroma had a significantly worse cumulative survival, compared to the patients with below-the-median macrophage number in the tumor stroma (P = 0.000, Figure 2c). When the ratio of the macrophage number in the tumor islets versus the macrophage number in the tumor stroma was calculated, the patients with above-the-median ratio (islets/stroma) had a significantly better cumulative survival, compared to the patients with below-the-median ratio (P = 0.000, Figure 2d).

**Figure 3:**
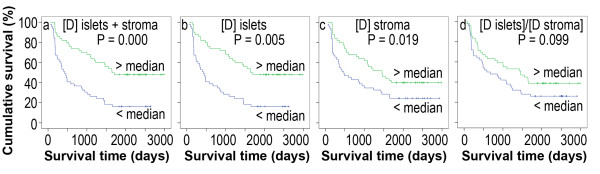
**Kaplan-Meier survival curves demonstrate mature dendritic cell number or ratio in correlation to survival**. [D] represents the mature dendritic cell number in the tumor islets and stroma (a), tumor islets (b), and tumor stroma (c). (d) The ratio of the mature dendritic cell number in the tumor islets "[D islets]" versus the mature dendritic cell number in the tumor stroma "[D stroma]".

The patients with above-the-median numbers of mature dendritic cells in the tumor islets and stroma, tumor islets, and tumor stroma had a significantly better cumulative survival, compared to the patients with below-the-median numbers of mature dendritic cells (P = 0.000, 0.005, and 0.019, respectively, Figure 3a-c). The patients with above-the-median ratio (islets/stroma) had a similar cumulative survival, compared to the patients with below-the-median ratio (P = 0.099, Figure 3d).

**Figure 4:**
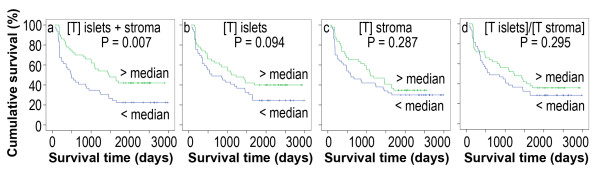
**Kaplan-Meier survival curves demonstrate cytotoxic T cell number or ratio in correlation to survival**. [T] represents the cytotoxic T cell number in the tumor islets and stroma (a), tumor islets (b), and tumor stroma (c). (d) The ratio of the cytotoxic T cell number in the tumor islets "[T islets]" versus the cytotoxic T cell number in the tumor stroma "[T stroma]".

The patients with above-the-median number of cytotoxic T cells in the tumor islets and stroma had a significantly better cumulative survival, compared to the patients with below-the-median number of cytotoxic T cells (P = 0.007, Figure 4a). There were no significant differences between the above-the-median and below-the-median groups, in regard to the number of cytotoxic T cells in the tumor islets or tumor stroma, or the ratio of the cell number in the tumor islets versus the cell number in stroma (P = 0.094, 0.287, and 0.295, respectively, Figure 4b-d).

In order to determine whether the number of tumor-associated immune cells is correlated to the clinicopathological characteristics, the number of cases with < median cell number was compared with the number of cases with ≥ median cell number. We found that the number of macrophages was not correlated with the tumor stage, tumor grade, or lymph node metastasis, except that the adenocarcinomas had more macrophages in the tumor islets and stroma, compared to the non-adenocarcinomas (i.e., squamous and large cell carcinomas) (P = 0.012) (Table 3). However, there were no significant differences when the numbers of macrophages in the tumor islets only or tumor stroma only were compared. The number of mature dendritic cells was less in the tumors of advanced stages (III-IV) or with lymph node metastasis, compared to those of early stages (I-II) or without lymph node metastasis (Table 4). In addition, there were more mature dendritic cells in the tumor islets and stroma of the non-adenocarcinomas, compared to the adenocarcinomas (Table 4). In contrast, the number of cytotoxic T cells was not correlated with the tumor stage, histology, or lymph node metastasis, except that the tumors of poor differentiation had slightly more cytotoxic T cells in the tumor islets (Table 5).

**Table 3:** Correlation between the number of macrophages and clinicopathological characteristics of patients

Variable	Islets + Stroma			Islets			Stroma		
	
	< median	≥ median	P	< median	≥ median	P	< median	≥ median	P
Tumor stage:									
I-II	27	28	0.929	24	31	0.228	31	24	0.158
III-IV	22	22		25	19		18	26	
Histology									
Adenocarcinoma	16	29	0.012	19	26	0.228	19	26	0.228
Non-adenocarcinoma	33	21		30	24		30	24	
Tumor grade									
Well to moderate differentiation	26	24	0.646	26	24	0.248	26	24	0.495
Poor differentiation	14	16		11	19		13	17	
Lymph node metastasis									
No	28	31	0.624	25	34	0.103	33	26	0.153
Yes	21	19		24	16		16	24	

**Table 4:** Correlation between the number of mature dendritic cells and clinicopathological characteristics of patients

Variable	Islets + Stroma			Islets			Stroma		
	
	< median	≥ median	P	< median	≥ median	P	< median	≥ median	P
Tumor stage:									
I-II	21	34	0.012	23	32	0.107	22	33	0.044
III-IV	28	16		26	18		27	17	
Histology									
Adenocarcinoma	30	15	0.002	27	18	0.070	27	18	0.070
Non-adenocarcinoma	19	35		22	32		22	32	
Tumor grade									
Well to moderate differentiation	21	29	0.861	23	27	0.647	26	24	0.066
Poor differentiation	12	18		12	18		9	21	
Lymph node metastasis									
No	23	36	0.014	24	35	0.041	25	34	0.103
Yes	26	14		25	15		24	16	

**Table 5:** Correlation between the number of cytotoxic T cells and clinicopathological characteristics of patients

Variable	Islets + Stroma			Islets			Stroma		
	
	< median	≥ median	P	< median	≥ median	P	< median	≥ median	P
Tumor stage:									
I-II	23	32	0.107	24	31	0.228	27	28	0.840
III-IV	26	18		25	19		23	21	
Histology									
Adenocarcinoma	24	21	0.547	23	22	0.841	24	21	0.688
Non-adenocarcinoma	25	29		26	28		26	28	
Tumor grade									
Well to moderate differentiation	26	24	0.248	29	21	0.040	28	22	0.248
Poor differentiation	11	19		10	20		12	18	
Lymph node metastasis									
No	25	34	0.103	27	32	0.416	29	30	0.838
Yes	24	16		22	18		21	19	

In order to determine whether the number of tumor-associated immune cells is independently associated with patient's survival time, the multivariate Cox proportional hazards analysis was used. The tumor stage, histology, grade and lymph node metastasis were included in the multivariate analysis along with the cell numbers. We found that only the macrophage numbers in the tumor islets or tumor stroma were independent predictors of patient's survival time (P = 0.028 or 0.002, respectively). The numbers of mature dendritic cells or cytotoxic T cells were not independent prognostic factors (Table 6).

**Table 6:** Correlation between the number and microlocalization of immune cells and patient's survival time by a multivariate Cox proportional hazards model analysis

Factor	Hazard ratio	95% Confidence interval	P
Tumor stage	6.790	1.319 - 34.952	0.022
Histology	0.973	0.934 - 1.003	0.116
Tumor grade	0.208	0.076 - 2.669	0.228
Lymph node metastasis	0.661	0.260 - 1.679	0.384
Macrophages in islets	0.909	0.834 - 0.990	0.028
Macrophages in stroma	1.080	1.029 - 1.133	0.002
Ratio of islets/stroma of macrophages	0.970	0.847 - 1.111	0.663
Mature dendritic cells in islets and stroma	1.061	0.938 - 1.200	0.350
Mature dendritic cells in islets	0.922	0.841 - 1.012	0.087
Mature dendritic cells in stroma	1.002	0.933 - 1.075	0.963
CD8+ T cells in islets and stroma	1.013	0.988 - 1.039	0.311
CD8+ T cells in islets	1.029	0.992 - 1.068	0.125
CD8+ T cells in stroma	0.978	0.934 - 1.023	0.335

## Discussion

Macrophage is a major component of inflammatory infiltrate of tumors [8,9]. Studies on the tumor-associated macrophages (TAMs) in non-small cell lung cancer have reported controversial results. For example, Chen et al reported that the TAMs are negatively associated with survival in the NSCLC patients [10]. Toomey et al found that there is no association between the macrophage number and prognosis of NSCLC [11]. On the other hand, Welsh et al found that the macrophage density in the tumor islets is positively associated with patient's survival [12]. Our previous study showed that the macrophage number in the tumor islets is positively associated with survival, whereas the macrophage number in the tumor stroma is negatively associated with survival [6]. In this study, we again confirmed our previous findings. In addition, we found that the total number of macrophages in both the tumor islets and stroma does not predict prognosis. This may be due to the fact that the macrophage number in the tumor islets is positively associated with survival, whereas the macrophage number in the tumor stroma is negatively associated with survival. Therefore, the positive and negative associations antagonize each other and reduce the net predictive value when the two macrophage pools are counted together. On the other hand, the ratio of macrophage number in the tumor islets versus the macrophage number in the tumor stroma appears to better predict the cumulative survival in the Kaplan-Meier survival curves and univariate analysis (Figure 2d). However, this ratio is not an independent predictor of patient's survival in the multivariate analysis (Table 6). We speculate that this is because some clinicopathological characteristics may affect the distribution of the ratio and thus its statistic significance in the multivariate analysis. For example, the overall macrophage number per high-power field was increased in the adenocarcinomas, which may have an effect on the predictive value of macrophage ratio in the tumor islets/stroma. The puzzle of why the macrophages in the tumor islets are good for prognosis but the macrophages in the tumor stroma are bad for prognosis may be solved by investigating microlocalization of the M1 and M2 forms of TAMs. The M1 macrophages have anti-tumor functions, whereas the M2 macrophages contain pro-tumor functions [13,14]. A recent study demonstrated that 70% of macrophages in the tumor islets are the M1 macrophages in the NSCLC patients with extended survival [15]. We recently found that about 85% of the macrophages in the tumor stroma are the M2 macrophages in the NSCLC patients with an average of 1-year survival (Ma, et al., unpublished observation).

The density of tumor-infiltrating mature dendritic cells has been found to be a better predictor of clinical outcome than other parameters in a recent study [16]. Our results are consistent with this report. The predictive value of the numbers of mature dendritic cells is only significant in the univariate analysis but not in the multivariate analysis. This implies that we need to be cautious in using the number of mature dendritic cells to predict the patient's outcome.

The number of CD8+ T cells in the tumor islets is found to be positively associated with survival time in patients with the stage IV NSCLC [17]. Our study shows the same trend but fails to reach any statistical significance (Figure 4b, P = 0.094). We reason that this is because our patient population (N = 99) is smaller than that study's patient population (N = 199) [17]. Both studies showed that the number of cytotoxic T cells in the tumor stroma is not associated with patient's survival time. In our study, we found that the total number of cytotoxic T cells in both the tumor islets and stroma is positively associated with patient's survival time in the univariate analysis but not in the multivariate analysis. This suggests that the clinical significance of counting the number of cytotoxic T cells is limited, compared to counting the number of macrophages in the tumor islets or stroma. The observed differences among the three immune cells may be caused by the differences in their biological functions. Macrophages not only can serve as antigen-presenting cells (like mature dendritic cells), but also can serve as cytotoxic cells (like cytotoxic T cells) through releasing reactive oxygen/nitrogen intermediates and TNFα to kill the nearby tumor cells [18].

In the present study, the 5-year survival rate is 44% for the patients without lymph node metastasis and 15% for the patients with lymph node metastasis (Table 1), the difference of which is very significant (P = 0.002, χ^2 ^analysis). However, it is surprising that the status of lymph node metastasis is not associated with patient's survival in a multivariate Cox proportional hazards model analysis (Table 6). Other studies have shown that lymph node metastasis is an independent prognostic factor in multivariate analysis in patients with NSCLC [19,20]. We speculate that the main reason for this discrepancy is that the sample size in our series is not big enough to reach statistical significance in the multivariate analysis. This speculation may also be applied to the association of the density of mature dentritic cells or cytotoxic T cells with the survival in the multivariate analysis, because it is possible that these immune parameters would reach statistical significance in a bigger cohort of patients.

The limitation of this study is that each immune cell type is identified by a single marker. CD68, CD83 and CD8 are widely used to identify macrophages, mature dendritic cells and cytotoxic T cells, respectively [15,17,21-25]. However, these markers are also expressed by other cell types. For example, CD68 has been found in immature CD1a-positive dendritic cells [26,27]. CD83 is also expressed by other cell types such as B lymphocytes [28]. A better marker for mature dendritic cells is DC-Lamp (CD208, using rat IgG2a antibodies, clone SR1010.E1, from Schering-Plough, Kenilworth, NJ) for paraffin-embedded tissue sections [16]. Although most tumor-infiltrating CD8+ T cells are cytotoxic T cells [25], some CD8+ T cells are regulatory T cells [29]. Therefore, it is possible that a portion of the immune cells identified by these markers are not the named immune cells (i.e., macrophages, mature dendritic cells, and cytotoxic T cells, respectively).

## Conclusions

This study demonstrates that macrophages, mature dendritic cells, and cytotoxic T cells are present more in the tumor stroma than in the tumor islets in non-small cell lung cancer. Although the number and microlocalization of the three tumor-associated immune cells appear similar, the clinical significance of counting these cells is quite different. The tumor-associated macrophages may be the most important immune cells in NSCLC, because the number of TAMs in the tumor islets or stroma can independently predict patient's survival time.

## Competing interests

The authors declare that they have no competing interests.

## Authors' contributions

FD performed immunohistochemistry, evaluated the stained slides, and performed statistical analysis. LL and GC designed and supervised the collection of data. NY collected the clinicopathological data. QP, SZ, JM, and LM participated in making the slides, evaluation of the stained slides, and data analysis. ZY analyzed and interpreted the data and prepared the tables, figures, and manuscript text. All authors participated in manuscript preparation and approved the final version prior to submission.

## Pre-publication history

The pre-publication history for this paper can be accessed here:

http://www.biomedcentral.com/1471-2407/10/220/prepub
